# Ketogenic nutritional therapy (KeNuT)—a multi-step dietary model with meal replacements for the management of obesity and its related metabolic disorders: a consensus statement from the working group of the Club of the Italian Society of Endocrinology (SIE)—diet therapies in endocrinology and metabolism

**DOI:** 10.1007/s40618-023-02258-2

**Published:** 2024-01-18

**Authors:** L. Barrea, M. Caprio, E. Camajani, L. Verde, S. Perrini, A. Cignarelli, F. Prodam, A. Gambineri, A. M. Isidori, A. Colao, F. Giorgino, G. Aimaretti, G. Muscogiuri

**Affiliations:** 1 Dipartimento di Scienze Umanistiche, Università Telematica Pegaso, Centro Direzionale, Via Porzio Isola F2, 80143 Naples, Italy; 2 Laboratory of Cardiovascular Endocrinology, IRCCS San Raffaele, Rome, Italy; 3 Department of Human Sciences and Promotion of the Quality of Life, San Raffaele Roma Open University, 00166 Rome, Italy; 4 Department of Public Health, University “Federico II” of Naples, 80138 Naples, Italy; 5 Department of Precision and Regenerative Medicine and Ionian Area, Section of Internal Medicine, Endocrinology, Andrology and Metabolic Diseases, University of Bari Aldo Moro, Bari, Italy; 6 Endocrinology, Department of Translational Medicine, Università del Piemonte Orientale, 28100 Novara, Italy; 7 Department of Health Sciences, University of Piemonte Orientale, 28100 Novara, Italy; 8 Division of Endocrinology and Diabetes Prevention and Care, IRCCS Azienda Ospedaliero-Universitaria di Bologna, Bologna, Italy; 9 Department of Medical and Surgical Sciences (DIMEC), Alma Mater Studiorum University of Bologna, Bologna, Italy; 10 Department of Experimental Medicine, Sapienza University of Rome, Viale Regina Elena 324, 00161 Rome, Italy; 11 Dipartimento di Medicina Clinica e Chirurgia, Endocrinologia, Unità di Diabetologia e Andrologia, Università degli Studi di Napoli Federico II, Via Sergio Pansini 5, 80131 Naples, Italy; 12 Centro Italiano per la Cura e il Benessere del Paziente con Obesità (C.I.B.O), Unità di Endocrinologia, Diabetologia e Andrologia, Dipartimento di Medicina Clinica e Chirurgia, Università degli Studi di Napoli Federico II, Naples, Italy; 13 Cattedra Unesco “Educazione Alla Salute e Allo Sviluppo Sostenibile”, Università degli Studi di Napoli Federico II, Naples, Italy

**Keywords:** Ketogenic diet, VLCKD, Meal replacements, Ketone bodies, Weight loss, Cardiovascular risk, Type 2 diabetes, Cardiovascular rehabilitation

## Abstract

**Purpose:**

The ketogenic nutritional therapy (KeNuT) is an effective dietary treatment for patients with obesity and obesity-related comorbidities, including type 2 diabetes, dyslipidaemia, hypertension, coronary artery disease, and some type of cancers. However, to date an official document on the correct prescription of the ketogenic diet, validated by authoritative societies in nutrition or endocrine sciences, is missing. It is important to emphasize that the ketogenic nutritional therapy requires proper medical supervision for patient selection, due to the complex biochemical implications of ketosis and the need for a strict therapeutic compliance, and an experienced nutritionist for proper personalization of the whole nutritional protocol.

**Methods:**

This practical guide provides an update of main clinical indications and contraindications of ketogenic nutritional therapy with meal replacements and its mechanisms of action. In addition, the various phases of the protocol involving meal replacements, its monitoring, clinical management and potential side effects, are also discussed.

**Conclusion:**

This practical guide will help the healthcare provider to acquire the necessary skills to provide a comprehensive care of patients with overweight, obesity and obesity-related diseases, using a multistep ketogenic dietary treatment, recognized by the Club of the Italian Society of Endocrinology (SIE)—Diet Therapies in Endocrinology and Metabolism.

## Introduction

In the last 10 years, ketogenic nutritional therapy gained great interest in the context of the management of patients with overweight or obesity [1], as well as with different emerging clinical settings, including psoriasis [2, 3], cancer [4, 5], sleep disorders [6, 7], male hypogonadism [8] and polycystic ovary syndrome (PCOS) [9–13]. Due to the different clinical studies published on this topic, the Italian Society of Endocrinology (SIE) recently published a consensus statement [14]. Several ketogenic diets with different macronutrients composition exist, including the classic ketogenic diet, the Atkins diet, the high-fat ketogenic diet, and the very-low-calorie ketogenic diet (VLCKD). To clarify the confusion potentially raised by the complex nomenclature of ketogenic diets, Trimboli et al. [15] recently published a document in which a clear definition, classification and acronym of various ketogenic diets has been reported, as shown in Table 1.

**Table 1:** Definition, classification, and acronym of various ketogenic diets

Diet	Acronym	Kcal	Carbohydrates	Lipids
Isocaloric ketogenic diet	ICKD	Calculated according to TEE	< 30–50 g/day	70–80% of DCI
Low-calorie ketogenic diet	LCKD	> 800 kcal/day but < TEE	< 30–50 g/day	> 30–40 g/day
Very low-calorie ketogenic diet	VLCKD	< 800 kcal/day	< 30–50 g/day	< 30–40 g/day

*ICKD* isocaloric ketogenic diet; *TEE* total energy expenditure; *LCKD* low-calorie ketogenic diet; *VLCKD* very low-calorie ketogenic diet; *DCI* daily calorie intake

In general, to be defined “ketogenic”, the diet should not exceed a total amount of carbohydrate higher than 30–50 g/day. Glucose production in the first 3 to 4 days is provided by gluconeogenesis and glycogenolysis. Lowering glucose levels will lead to lower insulin levels and increased glucagon levels. This hormonal change induces free fatty acids release from the adipose tissue which, arriving via the bloodstream to the liver, will be transformed into ketone bodies as an alternative fuel source for extra hepatic organs as central nervous system, kidney, muscle, and heart. Ketogenic diets induce a metabolic condition called “nutritional ketosis”, which is completely different from the pathologic state of diabetic ketoacidosis [16]. The restriction of carbohydrate consumption to produce ketone bodies determines a nutritional ketosis whose hallmark is characterized by a blood ketone level between 0.5 and 3 mg/dL. On the other hand, “therapeutic ketosis”, requires a concentration of ketones greater than 5 mg/dL but never exceeding 8–9 mg/dL [17]; this type of ketosis is considered more effective than nutritional ketosis. Interestingly, a few weeks after nutritional (or therapeutic) ketosis, a process known as “keto-adaptation” occurs, which represents the cells’ ability to adapt and respond primarily  to ketones bodies as fuel source, instead of glucose [16]. This process is due to the up-regulated transcription of genes encoding metabolic enzymes leading to increased mitochondrial density in oxidative tissues such as muscle and brain [16]. Differently, in the pathologic state of diabetic ketoacidosis plasma ketones concentrations is five- to tenfold greater than the levels observed during nutritional ketosis [16]. Indeed, despite similar names, nutritional (or therapeutic) ketosis represents a completely different metabolic process. The production of endogenous insulin naturally occurring in nutritional (or therapeutic) ketosis is protective against the occurrence of diabetic ketoacidosis [16]. Additionally, in nutritional (or therapeutic) ketosis the body is able to maintain both a normal pH and normal blood glucose levels, whereas abnormal pH and extremely elevated blood glucose are observed in diabetic ketoacidosis [16].

VLCKD is characterized by a reduced calorie intake, usually less than 800 kilocalories; on the other hand, in isocaloric-ketogenic diet and low-calorie ketogenic diet it is not possible to define the caloric content a priori, given that the definition of isocaloric and hypocaloric is established on the total energy expenditure of individual patients [18–20]. From a macronutrient point of view, VLCKD provides approximately 0.8–1.2 g/day of protein per each kg of ideal body weight and 20–30 g/day of fat [21].

The benefits of VLCKD on body weight, body composition, glucose and lipid parameters have been recently demonstrated in the European guidelines for obesity management in adults [1]. Authors reported that VLCKD induced a significant weight loss in the short, intermediate, and long term and determined an improvement in glucose and lipid profiles as well as in body composition parameters. In addition, VLCKD compared to other weight loss dietary interventions of the same duration, showed a higher efficacy in the reduction of body weight and in particular of waist circumference and fat mass, as well as in the improvement of insulin resistance, total cholesterol and triglyceridemia [1]. Importantly, reduction in body weight during VLCKD occurs by preserving lean mass and by selectively burning fat in visceral rather than subcutaneous adipose tissue compartments, thus optimizing body composition [22–25]. Another metabolic advantage of VLCKD is the preservation of energy expenditure, thus preserving energy balance with a consequent facilitation to weight loss [1]. Under physiological conditions, prolonged caloric restriction triggers biological responses that result in decreased energy expenditure, inducing resistance to weight loss [26]. By contrast, it was observed that VLCKD did not foster the expected reduction in energy expenditure in patients with obesity, despite the large body weight reduction [27]. It could be hypothesized that nutritional ketosis during VLCKD could promote energy expenditure by increasing the expression of thermogenic genes, a biological process that dissipates energy through futile metabolic cycles, counteracting metabolic inflexibility typical of obesity [28–31]. VLCKD benefits go beyond weight loss, improving physical and sexual activity, sleep disturbances, and reducing food cravings, thus ameliorating the quality of life [32]. Moreover, it is known that VLCKD could improve several obesity- and overweight-associated comorbidities, such as type 2 diabetes (T2DM), hypogonadism, PCOS, respiratory dysfunctions, and cardiovascular diseases [27]. This psychological well-being, combined with the improved perception of personal fitness, encourages patients’ adherence to the diet and strongly contributes to the success of the weight-loss intervention [32]. Of interest, both the EASO guidelines [1] and a recent article by Barrea et al. [33] report that the VLCKD can be considered a safe dietary approach since the most common side effects are easy to be managed and usually clinically mild.

Meal replacements are often used in VLCKD protocols and include portion-controlled, ready-made meals and products marketed as bars, shakes, and soups. In order to reduce the daily calorie intake, meal replacements are used instead of fresh food [34]. Using meal replacements has the advantage of being helpful for calorie control, since patients often tend to overestimate the amount of calories in food [35], and self-efficacy for dietary behaviours is known to decline over time in behavioural intervention [36]. Importantly, a large variety of stimuli in a given meal is usually associated with higher calorie intake, and a meal replacement narrows all sensory stimuli by providing a limited range of flavours, textures and macronutrient within and between eating episodes [37]; therefore, properly formulated meal replacements have the ability to decrease hedonic drive for food and increases satiety. A recent systematic review by Astbury et al. [38] reported that dietary therapies incorporating meal replacements, in comparison to weight loss programmes without meal replacements, were associated with greater weight loss (a mean difference of −2.22 to −6.13 kg) at 1 year. In this context, meal replacements should be considered as a valid option for management of overweight and obesity [38].

In the recent Italian and European position statements, VLCKDs have been proposed as a valuable option to achieve significant weight loss in a short period of time. However, their long-term benefits in treating obesity require more robust evidence given that most studies evaluated only short- and medium-term effects. Therefore, VLCKD represents a medicalized nutritional therapy in the context of a more complex, multidimensional approach for the long-term management of patients with obesity, also including consideration of behavioural, pharmacological and surgical treatment options [39].

The purpose of this practical guide from the working group of the Club of the SIE society—Diet Therapies in Endocrinology and Metabolism is to provide a useful manual for clinicians and nutritionists, aimed at the correct prescription of a multistep VLCKD protocol with meal replacements, followed by a transition to a Mediterranean-based diet combined to a physical activity program, for patients with overweight and obesity.

## VLCKD: indications and contraindications

The prescription of VLCKD should be tailored according to individuals’ characteristics of single patient, considering the reported indications or contraindications (Table 2).

**Table 2:** Indications and contraindications for VLCKD prescription

Indications	Contraindications
Obesity (BMI ≥ 30.0 kg/m^2^)	Pregnancy and breastfeeding
Obesity with one or more comorbidities (T2DM^a^, locomotors system disorder, NAFLD)	Childhood^b^
Overweight (BMI = 25.0–29.9 kg/m^2^) with abdominal obesity (waist circumference > 102 cm in men and > 88 cm in women)	Rare metabolic disorders (porphyria, carnitine deficiency, carnitine palmitoyltransferase deficiency, carnitine-acylcarnitine translocase deficiency, mitochondrial fatty acid β-oxidation disorders, and pyruvate carboxylase deficiency)
Overweight/obesity, PCOS and hypogonadism	Type 1 diabetes, type 2 diabetes with beta cell failure or concomitant treatment with SGLT2 inhibitors
Weight loss before bariatric surgery or in case of weight regain after bariatric surgery	Organ failure (liver, kidney, or heart NYHA III e IV)
Overweight/obesity and neurological/neurodegenerative disorders (Parkinson, Alzheimer, amyotrophic lateral sclerosis, epilepsy)	Recent myocardial infarction or cerebrovascular stroke
Overweight/obesity and pulmonary dysfunctions (OSAS, COPD, OHS, asthma)	Severe psychiatric disorders
Overweight/obesity and psoriasis	Severe eating disorders
	Alcohol and substance abuse

*T2DM* type 2 diabetes mellitus; *NAFLD* non-alcoholic fatty liver disease; *OSAS* obstructive sleep apnoea syndrome; *PCOS* polycystic ovary syndrome; *OSAS* obstructive sleep apnoea syndrome; *COPD* chronic obstructive pulmonary diseases; *OHS* obesity hypoventilation syndrome

^a^Use of sodium/glucose cotransporter 2 (SGLT2) inhibitors or insulin therapy is forbidden

^b^During childhood VLCKD could be prescribed in particular condition (epilepsy, high insulin resistance)

Short- and medium-term studies support the use of VLCKD in individuals with obesity (body mass index, BMI ≥ 30.0 kg/m^2^) or in overweight (BMI 25.0–29.9 kg/m^2^) with abdominal obesity (waist circumference > 102 cm in men and > 88 cm in women). In addition, VLCKD can be recommended when the excess body weight is associated with complications, i.e., T2DM, locomotor system disorders, non-alcoholic fatty liver disease (NAFLD), cardiovascular diseases [1, 14], PCOS [9, 10, 14], and hypogonadism [8].

Moreover, VLCKD is strongly recommended to achieve weight loss before bariatric surgery or in case of weight regain after the intervention [40–42]. Increasing evidence also suggests that VLCKD could positively affect respiratory function thus improving obstructive sleep apnoea syndrome (OSAS), asthma, obesity hypoventilation syndrome (OHS) and chronic obstructive respiratory diseases (COPD) [43–45]. Finally, several studies have shown that nutritional ketosis could improve brain function in neurological and neurodegenerative disorders (epilepsy, Parkinson, Alzheimer and sleep disorders) [46, 47], and reduce inflammation in psoriasis and respiratory infections [3, 48].

Conversely, some conditions represent absolute contraindications to the use of VLCKD [1, 14, 49]. In particular, pregnancy and breastfeeding,  as well as childhood are physiological conditions during which VLCKD should be avoided. Pathological conditions contraindicating VLCKD include chronic kidney disease, type 1 diabetes mellitus, T2DM with beta cell failure or concomitant treatment with sodium-glucose co-transporter 2 inhibitors (SGLT2i), rare metabolic disorders (porphyria, carnitine deficiency, carnitine palmitoyltransferase deficiency, carnitine-acylcarnitine translocase deficiency, mitochondrial fatty acid β-oxidation disorders, and pyruvate carboxylase deficiency), recent myocardial infarction or cerebrovascular stroke, severe psychiatric disorders. In addition, although VLCKD could improve several obesity-related diseases that affect liver, heart and lungs, there are still no sufficient data to recommend this treatment in patients with organ failure or with NYHA class III or higher. Moreover, eating disorders, alcohol and substance abuse also represent contraindications to the use of VLCKD [1, 14, 49].

Surprisingly, the recent European recommendations for the dietary management of diabetes” by the Diabetes and Nutrition Study Group (DNSG) of the EASD, recently published in Diabetologia, recommended against the use of ketogenic diets for weight loss in patients with diabetes [50]. This recommendation was based on potential safety concerns, including increased low-density lipoprotein cholesterol levels, hypoglycaemia, ketoacidosis, and vitamin and mineral inadequacies, but these observations were mainly based on case reports of unrecognised autoimmune diabetes, T2DM treated with a SGLT2i, alcohol use or breastfeeding, all conditions that clearly contraindicate the use of a VLCKD [51].

For all these reasons, it is important to recall that the ketogenic nutritional therapy (KeNuT) requires a strict medical supervision, consisting in a detailed anamnesis, physical examination and interpretation of blood and urine parameters, as well as assessment and eventual modification of pharmacological therapy. The role of the nutritionist, on the other hand, is essential for the correct setting of VLCKD, assessment of allergies and/or intolerances, customizing protein requirements, and guidance of the patient during carbohydrate reintroduction and increase of calorie content, until and during the maintenance diet.

## VLCKD with meal replacement

### The biochemical mechanisms of VLCKD

The main goal of VLCKD is to inhibit lipogenesis and promote the burning of fat storage. Because of metabolic flexibility, humans can rely on alternative sources of energy, depending on their availability [52]. Therefore, the restriction of carbohydrate intake (30–50 g/day) prompts the body to metabolically shift to nutritional ketosis, which relies on the hepatic production of ketones that provide energy to almost all cells in the human body [16]. These metabolic modifications are triggered by hormonal changes. First, a lower carbohydrate intake reduces insulin secretion, thus inhibiting glycogen and lipid synthesis. On the other hand, glucagon concentration is increased with a reversal of insulin/glucagon ratio. Glucagon is a lipolytic hormone targeting the catabolism of triglycerides in the adipose tissue. Thus, the breakdown of triglycerides provides free fatty acids and glycerol which is converted into glucose by hepatic gluconeogenesis. This process contributes to the homeostasis of blood glucose concentrations during the ketogenic diet. In addition, free fatty acids are transformed into ketone bodies (acetoacetate, β-hydroxybutyrate, and acetone) by β-oxidation in the liver. During VLCKD, nutritional ketosis is stable (blood ketone levels of 0.5–3 mg/dL) by virtue of mutual control between ketone bodies and insulin concentrations. Ketones can be used as energy source for the brain, renal cortex, heart, and skeletal muscles. In particular, the utilization of ketones as an alternative fuel source in skeletal muscles might preserve sparing lean muscle mass from gluconeogenesis, whereby glucose is obtained from amino acids [16]. Moreover, an adequate protein intake provided by meal replacement can contribute to maintaining constant insulin levels (insulinotropic effect) and promote the secretion of growth hormone (GH), a protein-sparing anabolic hormone, thus enhancing fat loss while preserving lean body mass and muscle [53]. Importantly, this body reassessment occurs through a reduction in adiposity, which mainly targets visceral fat, which is known to be more harmful than subcutaneous fat [22]. Indeed, treatment of patients with obesity with VLCKD modulates also food intake regulatory mechanisms by increasing serum levels of orexin-A, a neuropeptide synthesized in the lateral hypothalamus that reduces the sense of hunger and inhibits adipogenesis in abdominal visceral rather than subcutaneous adipocytes [22]. In addition, the ketogenic diet is also responsible for multiple beneficial effects beyond weight loss, preventing the development of several pathological conditions. Among these, neurodegenerative diseases, also provocatively named type 3 diabetes by some authors, are characterized by energy deficiency in neuronal cells, which could be improved by the shift to a ketone-based metabolism [54]. Ketones can replace glucose as the main fuel source for the brain, thus preventing the detrimental effects of impaired glucose metabolism in neuronal cells of patients affected by Alzheimer disease [54]. Moreover, chronic ketosis could be helpful for the treatment of epilepsy by increasing the expression of genes associated with mitochondrial biogenesis and function in neurons, thus enhancing energy production with anticonvulsant effects [55]. Another beneficial effect of VLCKD, when used in neurodegenerative diseases, consists in the effect of ketones in restoring the function of complex I in the mitochondrial chain, which is known to be impaired in motoneurons of patients with amyotrophic lateral sclerosis (ALS) [56]. In addition, the metabolism of ketones reduces the production of reactive oxygen species (ROS) which have a key role in the development of Parkinson disease by damaging dopaminergic neurons [57]. Alongside the metabolic benefits, the ketogenic diet exerts anti-inflammatory effects through immunomodulatory mechanisms. In the brain, it reduces microglial activation and neuroinflammation, thus ameliorating cognitive impairment [58]. Moreover, it promotes T cell responses in the lung [48] and reduction of systemic levels of inflammatory cytokines [59], thus protecting against respiratory infections [48] and psoriasis [60]. Despite other dietary interventions can also reduce systemic inflammation, a more relevant effect was observed in patients treated with VLCKD, thus suggesting an additional weight loss-independent anti-inflammatory effect, probably induced by ketone bodies production [59, 61, 62]. Considering the role of inflammation and of the innate immune system in the development of lung diseases during obesity [63, 64], it is not surprising that ketogenic diet can improve OSAS [45], COPD [65], and asthma [44]. The rapid and significant loss of adiposity, which occurs mainly at the abdominal visceral compartments as previously described, reduces the mechanical obstruction of the lung that occurs in patients with android obesity. These favourable effects on mechanical abdominal encumbrance, combined with the increase in the physiological ventilatory response to hypercarbia induced by ketosis, make the VLCKD particularly suitable for patients with OHS [43]. Recovery of respiratory function could be particularly desirable for those patients with sleep disorders, particularly for those who experience abrupt awakenings caused by OSAS. Moreover, VLCKD could also improve the regulation of sleep/wake behaviour by increasing serum levels of orexin-A, which has been shown to positively affect circadian rhythm [22].

### Use of meal replacements for VLCKD

As reported by the European Guidelines for Obesity Management in Adults, the VLCKD includes high-biological-value protein (deriving from milk, peas, whey and soy), artificial meals and natural foods [1]. VLCKD can employ meal replacements or conventional foods (meat, fish and eggs). Basciani et al. carried out a prospective pilot study comparing the efficacy and safety of 45 days of VLCKDs using meal replacement food based on whey or vegetable protein with conventional animal protein on metabolic parameters, body composition and the composition of gut microbiota in a population of patients with obesity and insulin resistance [66]. A significant reduction in body weight, both in the whey protein group and the vegetable protein group was recorded. A reduction in body weight was also observed in the animal protein group, although it did not reach statistical significance. Of note, an increase in blood urea nitrogen and uric acid and significant reduction of the estimated glomerular filtration rate compared to baseline value in the animal protein group was recorded [66]. Moreover, a significant reduction of *Firmicutes* and increase in *Bacteroidetes* with resultant improved *Firmicutes/Bacteroidetes* ratio was observed; in particular, whey protein and vegetable protein were more effective in reducing the percentage of *Firmicutes* than the animal protein diet. The authors concluded that VLCKDs based on whey or vegetable protein, contained in meals replacement, determine a safer metabolic profile and healthier microbiota composition than the animal protein option [66]. Hence, VLCKD with meal replacements based on whey and vegetable protein would therefore seem more appropriate for patients with obesity or cardiometabolic diseases. Scientific evidence suggests that the use of meal replacements in the first active ketogenic step would be preferable to ensure a safe effective use of VLCKD [66]. Furthermore, meal replacements are functional meals that provide a balanced amount of macro- and micro-nutrients. A high level of ingredient purification and high-quality standards ensure maximum assimilation of the nutrients provided.

Most of the meal replacements proposed for the ketogenic phases contain 15–18 g of protein of high biological value, with a carbohydrate content of no more than 3.5 g and a fat content of no more than 4 g [14]. Therefore, meal replacement provides the correct intake of macro- and micronutrients and, thanks to the availability of single-dose packages, help to ensure a controlled and easy intake.

## Ketogenic nutritional therapy: a multistep dietary model

### Ketogenic phases

**Fig. 1:**
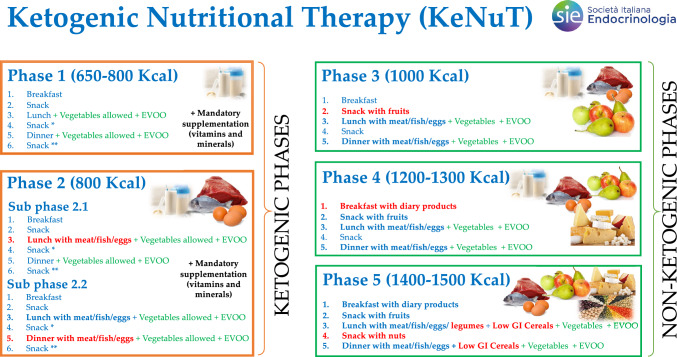
Schematic representation of the KeNuT multisteps dietary protocol with meal replacements proposed by the Club of the Italian Society of Endocrinology (SIE)—Diet Therapies in Endocrinology and Metabolism

The initial phase of VLCKD is characterized by a very low-calorie diet (650–800 kcal/day), low in carbohydrates (< 30 g daily from vegetables) and fat (only 20 g *per* day, derived from olive oil). The amount of high-biological-value protein ranged between 0.8 and 1.2 g *per* each kg of ideal body weight to preserve lean mass and to provide the minimal daily body requirements [21] (Fig. 1). During the visits of phase 1 and 2 (ketogenic phases) the healthcare provider should carry out clinical and nutritional evaluations, in particular: control of weight and body composition, measurement of blood pressure and evaluation of the main biochemical tests (renal, hepatic, electrolytes, and others at the physician's discretion). Assessment of capillary blood ketone levels could help monitoring of patient adherence and protocol efficacy.

#### Phase 1

In this phase, the patients eat high-biological-value protein meal replacements four or five times a day, according to the amount of protein needed. Of interest, it is important to use only low glycemic index/load and low-sugar vegetables to achieve the required amount of fibre, maintaining the low carbohydrate content. The dressings allowed in the active step are extra virgin olive oil (2 tablespoons in 24 h), flavourings and spices to taste, and lemon [1, 21]. Most common unintentional errors during this stage are systematic use of: drugs containing sugars, most often in the form of syrup or effervescent sachets, sugar-free candies and gums, herbal teas and teas with fruit pieces, balsamic vinegar, beverages from vending machines, even if sugar-free (tea and coffee), and sugar-free drinks. In this phase, supplementation with vitamins and mineral is mandatory since the diet is unbalanced and micronutrients are partly conveyed by food with a certain carbohydrate content. In particular, it is recommended to supplement patients with micronutrients (vitamins, such as complex B vitamins, vitamin C and E, minerals, including potassium, sodium, magnesium, calcium; and omega-3 fatty acids) according to international recommendations [1, 21], and summarized in Table 3. It is recommended to drink at least 2–2.5 L of water *per* day.

**Table 3:** Supplementation with micronutrients (vitamins, mineral salts and omega-3) recommended in active steps

	RDA^a^
Magnesium	240 mg
Potassium	3.9 g
Calcium	1000 mg
Vitamin C	105 mg
Vitamin E	13 mg
Selenium	55 μg
Vitamin A	700 μg
Vitamin B5	30 μg
Vitamin B6	1.3 mg
Vitamin B1	1.2 mg
Vitamin B2	1.6 mg
Folic acid	400 μg
Vitamin B12	2.4 μg
Vitamin D	15 μg
Omega-3	250 mg of EPA-DHA

*RDA* recommended daily allowance; *PRI* recommended intake of the population; *LARN* reference intake levels of nutrients and energy for the Italian population; *EPA* eicosapentaenoic acid; *DHA* docosahexaenoic acid

^a^RDA according to PRI of LARN adult between 18 and 60 years old

#### Phase 2

This VLCKD phase is similar to the previous one with a slight increase in calorie intake [1, 21]. In particular, a fresh protein meal is reintroduced to maintain nutritional ketosis: in fact, the purpose of this phase is to re-educate the patient to eat fresh protein, gradually eliminating meal replacements. The quantity of fresh natural proteins should be carefully established and personalized. Specifically, this phase is divided into two sub-phases: in the first, the patient replaces a single replacement meal (lunch or dinner) with fresh protein from meat, fish, and eggs, emphasizing that dairy products are still not allowed. In the second sub-phase, both lunch and dinner meals will be replaced with fresh protein. Also at this stage, it is important to remind the patient to use only low glycemic index and low sugar vegetables and to use only extra virgin olive oil (2 tablespoons per day), flavourings and spices, and lemon as dressings. It is still recommended to drink at least 2–2.5 L of water *per* day.

In this phase, only 2 or 3 meals replacement are maintained, for breakfast and snacks, always according to the protein content tailored to the individual patient. In these stages, the patient keeps maintaining a condition of nutritional ketosis, therefore supplementation with vitamins and minerals is always mandatory and the patient should be closely monitored. VLCKD phases are kept until the patient loses most of weight loss target, approximately 80%. For this reason, monitoring body composition is crucial [1, 21]. Accordingly, ketogenic phases may vary in duration, between 8 and 12 weeks, depending on individual characteristics and weight loss target. However, it should be emphasized that the duration of the ketogenic phases can be extended if the clinical conditions allow it, based on the patient's weight loss and adherence. In these ketogenic phases it is essential to examine the patient every 20–30 days. During the visit, it is important to monitor potential side effects, such as cramps, constipation, feeling of hunger, hypotension, headaches, menstrual cycle disorders, hair loss (mainly due to dehydration or non-adherence to food supplements, vitamins and minerals).

### Non-ketogenic phases

After the ketogenic phases, the patient is switched to a low-calorie, non-ketogenic diet (Fig. 1). At this point, the patients will progressively and slowly reintroduce different food groups [1, 21]. In particular, carbohydrates are gradually reintroduced, starting from food with the lowest glycaemic index (first fruit, then dairy products, legumes and finally carbohydrates with a higher glycemic index, such as cereals). Generally, the amount of carbohydrate *per* day in low-carbohydrate diets is approximately 60–130 g (≤ 20–45% of daily energy intake) [67].

#### Phase 3

This phase involves the reintroduction of fresh fruit as a snack. At breakfast (and possibly at the other snacks, always based on the personalized protein content on the individual patient) the patient should continue to consume a meal replacement. At lunch and dinner, as in phase 2, the patient consumes fresh proteins (meat, fish, eggs), the quantities thereof should be personalized for the individual patient. One portion of fruit per day, corresponding approximately to 150 g, as suggested by the Reference Intake Levels for Nutrients and Energy (Italian LARN, IV revision), is allowed. Cooked, syrupy, dried, dehydrated or juiced fruits are not recommended. In this phase, patients can eat all vegetables, cooked and raw. With the reintroduction of fruit, they can gradually interrupt the use of the vitamin and mineral supplements. The calories in this phase will be increased to approximately 1000 kcal. It is recommended to drink at least 2 L of water *per* day.

#### Phase 4

In this phase, low-fat dairy products are also reintroduced. The goal of this transitional step is to maintain the weight loss achieved in the previous phases and increase the average caloric intake, which will approximately reach 1200 kcal/day [1, 21]. The recommended dairy products are: skimmed or partially skimmed milk, low-fat cheese with less than 20% fat, and low-fat yogurt. In this phase, the intensification of physical activity to complete the re-education process for a healthy and active lifestyle, a prerequisite for maintaining the ideal weight over time, is fundamental. Also in this phase, all the cooked or raw vegetables can be eaten. At present, there are no specific indications on the length of this reintroduction step. It is recommended to drink at least 2 L of water *per* day. However, to physiologically restore a normal glucose metabolism without post-prandial hyperinsulinemia, a minimum duration of at least 21 days is recommended, before moving on to the next phase.

#### Phase 5

The fifth phase involves the use of a low-calorie diet, around 1400 kcal/day, with the introduction of legumes and whole grains. Recommended legumes include fresh legumes, such as beans, soy, broad beans, and peas or dried legumes such as chickpeas and lentils, and lupine. It is recommended to drink at least 2 L of water *per* day. Also in this phase, it is mandatory to intensify physical activity.

## Maintenance diet and physical activity

### Mediterranean diet

Once the slow reintroduction of all food classes has been completed, the last phase involves the return to a hypocaloric Mediterranean diet (HMD) (modified in the proportion of carbohydrates, which must be kept < 45% of total calories), where no meal replacements are maintained. Energy intake is tailored according to individual characteristics (age, weight, height, climate, physical activity, etc.). Usually, a maintenance regime contains average 1600–1700 kcal/day, introducing low glycaemic index cereals and pseudo-cereals. Energy intake is provided by 30% fat, 45% carbohydrate, and 25% protein. According to HMD, carbohydrate should be consumed as low-glycemic index food (i.e., whole grain-based products, and legumes) while sugar intake should be limited to less than 10% of total energy intake (TEI), avoiding sweets and sugar-sweetened beverages. In addition, dietary fibre intake should be increased (25–30 g/day) by consuming fibre-rich food, such as whole grain, legumes, fruit, vegetables, and nuts [68]. Total fat intake should be represented by more unsaturated fatty acids (monounsaturated and polyunsaturated fatty acids, 19% and 5% of TEI, respectively) than saturated fatty acids (9% of TEI) [68]. As for protein, plant protein (i.e., legumes and soy-derived products) should be increased while animal protein (fish, lean cuts of meat, eggs, and dairies) should be used as alternative options during the week [69]. More in detail, protein intake should be 1–1.2 g/kg of desirable weight (DW, i.e., weight corresponding to a BMI of 22.5 kg/m^2^). This nutritional profile can be obtained using a specific food frequency during the week [69], with:

- daily consumption of plant-based foods (fruits, vegetables, wholegrain, legumes, and nuts), and monounsaturated fat as the primary source of fat;
- moderate intake of animal protein and fat, mainly from fish and low-fat dairies, respectively;
- restricted consumption of sweets and processed food [70].

This nutritional profile allows patients to continue their nutritional re-education while maintaining the achieved weight loss. This last step with maintenance diet, through the acquisition of correct eating habits, is crucial for maintaining long-term results.

### Physical activity

Physical activity is the cornerstone of healthy living, combined with a healthy, balanced diet. In patients with overweight and obesity, an improved lifestyle is essential to achieve lasting weight loss. For this reason, the multistep protocol involves physical activity from the very first steps, with a gradual increase in intensity and type of exercise. In the ketogenic step, intense aerobic activity is highly counterproductive: the calorie deficit to which the patient is exposed would not guarantee sufficient energy and physical activity could lead to a depletion of the lean mass. Differently, it was showed that muscle mass is preserved when performing interval training (IT) [71, 72], and body composition is improved when IT is added to a VLCKD protocol [61]. Consequently, short cycles of muscle-strengthening exercises lasting up to 25–30 min twice a week are recommended at this stage, favouring bilateral and multi-joint exercises that include full dynamic movements. Exercises can be performed with overloads and/or free weights depending on the subject's training status, availability and personal preference [73]. In step 1, each session of physical exercise should be structured as follows: an initial part of warm up, breathing and stretching of the posterior chain, a second part based on functional exercises repeated for 20 s with a 10-s pause, a part of proprioception and balance, and finally a part focused on breathing. An example of the training session can be found in Table 4. In step 2, each session of physical exercise should be structured similarly to previous step as follows: an initial part of warm up, breathing and stretching of the posterior chain, a second part based on functional exercises repeated for 30 s with a 15 s pause, a part of proprioception and balance, and finally a part focused on breathing. An example of the training session can be found in Table 4. In the reintroduction step, a gradual increase in carbohydrate and daily calorie intake is recommended; hence, it is possible to increase physical activity with aerobic exercise. It is therefore suggested to add during the third step, to the simple basic rules of an active lifestyle and IT, a simple aerobic activity, such as a leisurely 30–40 min’ walk, at least twice a week (Table 4). The fourth and fifth steps are a gradual approach to the final daily routine and involve the inclusion of physical activity every day, alternating between intense aerobic activity and muscle build-up exercises. Most authoritative health guidelines state that 30–40 min of exercise a day are sufficient to keep our bodies active, prevent illness, maintain a balanced body composition and, most importantly, preserve a satisfactory and rewarding psycho-physical condition (Table 4).

**Table 4:** Recommended physical activity during VLCKD protocol

Sunday	Monday	Tuesday	Wednesday	Thursday	Friday	Saturday
**Phase 1 and 2**						
Walking	Rest	IT excercise	Rest	IT excercise	Rest	Rest
**Phase 3**						
Walking	Rest	IT excercise	Rest	IT excercise	Walking	Rest
**Phase 4 and Phase 5**						
Walking	Walking	IT excercise	Walking	IT excercise	Walking	Walking

*IT* interval training

### Follow-up

Regardless of the type of maintenance diet, patients should increase their nutritional awareness on a healthy diet and know how to manage their food choices to avoid weight regain. However, long-lasting changes of behaviour and lifestyle might be challenging. It is well known that obesity is a chronic, relapsing disease, and weight regain is frequent [74]. The proposed multistep dietary protocol paves the way to deep changes, but only represents the first step of a long path. Indeed, the nutritional and psychological support in the maintenance phase is a key element for a successful outcome. Patients should be supported to prevent relapse into wrong habits, lack of motivation, thus preventing weight regain and related disorders [74]. Therefore, a planned follow-up is strongly recommended. Regular visit should be scheduled every three months during the first year following the ketogenic step, and every 6 months thereafter. During the visit, nutritional and clinical assessment should be performed to evaluate nutritional and health status. Over dietary counselling, physical activity should be endorsed according to the current recommendations [74].

## Nutritional and clinical management of patients undergoing VLCKD

### Assessment of nutritional status

The assessment of nutritional status represents a pillar in the management of patients with obesity, in all stages of nutritional interventions. It consists in the assessment of dietary intake and the evaluation of body composition, which both provide important information to develop tailored interventions from the beginning of the protocol to long-term follow-up. Weight gain is related to energy imbalance that might be explained by increased energy intake, reduced energy expenditure, and alteration of body composition [75]. The assessment of dietary intake allows the estimation of usual dietary composition (energy, macronutrients, and other dietary components) but can also provide information about dietary habits and wrong behaviours. Therefore, it could allow the identification of key items that require changes to achieve long-lasting weight control, as well as to monitor patient’s adherence to the protocol. Different methods can be used for the purpose (24 h recall, dietary record, food frequency questionnaire, dietary history) but the choice depends on patients’ compliance [76].

On the other hand, the assessment of body composition can allow an objective evaluation of nutritional status. In addition, it can be used to monitor the effect of VLCKD or disease-related data (clinical features as well as prognostic information) [77]. Several methods can be used to evaluate body composition with different outcomes on precision and accuracy. Anthropometry is the easiest method to detect information on nutritional status. Body weight and height can be used to calculate BMI and provide an estimation of cardiometabolic risk linked to overweight/obesity. Indeed, the higher the BMI > 25.0 kg/m^2^, the increased the risk of cardiometabolic morbidity and mortality [78]. In addition, the assessment of waist circumference can give information about abdominal adiposity, which represents a risk factor for cardiovascular disease and T2DM [77, 79]. Nevertheless, it is worth mentioning that anthropometric measurements need standardized protocols to increase the accuracy of collected data as well as the reliability of repeated measurements [77]. In brief, height should be measured by a stadiometer with an accuracy of 0.5 cm, while body weight by a calibrated beam scale with an accuracy of 0.1 kg. During the measurements, patients should wear only light clothing and no shoes.

Waist circumference should be assessed by non-elastic tape on the naked abdomen looking on the side of the patient, about midway between the last rib and the iliac crest. Patients should stand with feet together, on a plane parallel to the ground, with their hands on hips. During the assessment, the patient should breathe normally, and the tape might not compress the skin and should be parallel to the floor.

Imaging techniques (dual-energy X-ray absorptiometry, computed tomography, magnetic resonance, and ultrasound scanning) are the most advanced methods to detect body composition. However, they are not even feasible in clinical practice since they are expensive, time-consuming, unavailable in some hospitals, or expose patients to radiation [80]. Although dual-energy X-ray absorptiometry should be the best choice, therefore, bioelectrical impedance (BIA) has been widely used in clinical practice and in research studies to assess body composition. The measurements should be performed under strictly standardized conditions (i.e., empty bladder). Although BIA does not directly measure body composition, it provides information about fat mass, free fat mass, and body water (total, intracellular and extracellular water) [75]. Therefore, BIA could give indirect information about the preservation of the lean body mass, i.e., muscle, and decrease of the fat mass during the VLCKD and in the follow-up, as well as dehydration, helping in suggesting the correct hydration. In particular, monitoring muscle mass could allow the early identification (and correction) of inadequate protein intake.

Of note, phase angle, a BIA-derived index, is an important tool used in various fields, including clinical nutrition, to assess and monitor nutritional status [81, 82]. It provides valuable insights into the body composition and cellular health of an individual. While it is not the sole indicator of nutritional status, it is considered a sensitive and informative measure in this context. It represents the relationship between resistance and reactance, which are components of electrical impedance. Phase angle is calculated as the inverse tangent of the ratio of reactance to resistance. In the context of nutritional status, the phase angle is influenced by several factors, including body cell mass, cell membrane integrity, fluid balance, and overall cellular health. A higher phase angle generally indicates better cell membrane function, increased cellular mass, and improved nutritional status. On the other hand, a lower phase angle may suggest cellular dysfunction, depletion of body cell mass, or certain health conditions. By monitoring the phase angle over time, healthcare professionals can gain insights into changes in body composition and cellular health. It can help assess the efficacy of nutritional interventions, such as dietary modifications or supplementation, and guide the adjustment of treatment plans accordingly [81, 82]. Interestingly, it has been reported that phase angle is an important parameter to be monitored during VLCKD [83]. Indeed, the phase angle can be used to identify inflammation in different clinical settings [84]. Recently, in a cohort of 260 women (aged 18–69 years, BMI 25.0–50.9 kg/m^2^) plasma C-reactive protein concentrations significantly decreased whereas phase angle increased after 31 days of the active stage of VLCKD. Interestingly, a significant inverse association between C-reactive protein and phase angle was observed, independently from confounding factors (BMI, waist circumference, age, and physical activity). Therefore, monitoring phase angle could be a useful tool to evaluate changes in the inflammatory status of VLCKD-treated patients [84].

### Biochemical and clinical parameters evaluation

Over the assessment of nutritional status, the evaluation of biochemical and clinical parameters should be performed at the beginning of the VLCKD and during the follow-up. Biochemical parameters should include metabolic parameters (glucose, insulin, glycated haemoglobin, and lipids), electrolytes, liver enzymes, renal function, and uric acid concentrations.

To assess and monitor insulin resistance, glucose and insulin concentrations can be used to calculate Homeostasis Model Assessment (HOMA-IR) [85]; in addition, blood pressure should be measured with an appropriate pressure cuff. As for anamnestic data, family history and the presence of obesity-related complications should be assessed (T2DM, hypertension, cardiovascular diseases, respiratory diseases, joint diseases, non-alcoholic fatty liver disease, sleep disorders, etc.) as well as other chronic diseases. In addition, patients should be asked for the presence of endocrine abnormalities (e.g., Cushing syndrome, thyroid diseases, etc.), eating disorders (overeating, night eating syndrome, binge eating, bulimia nervosa, etc.), depression, and other mood disorders.

The collection of data related to lifestyle (physical exercise, chronic stress, smoking cessation, etc.) could help to identify major flaws that need to be addressed to improve the efficacy of nutritional interventions in the long term. Finally, psychosocial factors, expectations, and motivation for change should be investigated.

### Safety of the ketogenic diet

Despite growing evidence on VLCKD efficacy, many concerns about the possible side effects still exist. A recent meta-analysis of 12 clinical trials (*n* = 801 middle-aged individuals; BMI 40.7 ± 8.9 kg/m^2^) reported the beneficial effects of VLCKD on hypertension, dyslipidemia, and T2DM [19]. However, a significant increase in serum sodium concentrations was reported, likely due to the relevant fluid loss that occurs during the VLCKD [19]. Other frequently reported symptoms include headache, asthenia, muscle pain and weakness, halitosis, and gastrointestinal symptoms (nausea, vomiting, constipation, diarrhoea) [19, 33, 86, 87]. In addition, some patients reported hypotension, hypoglycaemia, hyperuricemia, visual disturbances, urolithiasis, gallbladder disease, and hair loss [19, 33, 86, 87]. Nevertheless, most of the studies reported that VLCKD is well tolerated by patients with obesity, with or without T2DM [33, 87], as well as in children and adolescents [86]. Although safety long-term studies are still scarce, it has been reported that 1-year treatment with VLCKD in 15 patients with obesity and T2DM was safe and increased quality of life more as compared to a standard low-calorie diet [88]. However, patients might experience mild symptoms with a short duration (4–22 days) that did not require the interruption of the dietary protocol or medical intervention [33, 86, 87]. Dehydration-related disorders (i.e., dry mouth, headache, asthenia, hypotension, lethargy, and visual disturbances) can be easily avoided or treated by an adequate water intake (more than 2 L/day) and oral supplementation with vitamins (complex B vitamins, vitamins C and E) and minerals (potassium, sodium, magnesium, calcium). BIA could help in monitoring the hydration of patients. In addition, patients could use specific supplements (i.e., omega 3-fatty acids, probiotics) or drugs (osmotic laxative for severe constipation, allopurinol for hyperuricemia, ursodeoxycholic acid in the presence of gallstones [89]), if needed [33]. Therefore, KeNuT represents an effective and safe intervention to be proposed for properly selected patients to treat obesity and its related complications. However, it is worth mentioning that KeNuT should be prescribed under strict medical supervision with careful clinical and laboratory monitoring.

## Conclusions

In this practical guide of the working group of the Club of the Italian Society of Endocrinology (SIE)—Diet Therapies in Endocrinology and Metabolism, we report a useful guideline of the multistep KeNuT for the management of obesity and its related metabolic disorders (Fig. 1). KeNuT should be recommended as an effective dietary treatment for individuals with obesity, particularly for patients with severe obesity and/or comorbidities (such as inflammatory, metabolic and cardiovascular diseases) who need rapid and substantial weight loss. Thus, KeNuT should be prescribed only after a proper clinical assessment by the endocrinologist. In conclusion, this practical guide will help the healthcare provider to acquire the necessary skills to provide a comprehensive care of patients with overweight, obesity and obesity-related diseases, to maintain weight loss in the long term, reduce the risk for complication, improve the outcome of cardiovascular and metabolic rehabilitation, and finally ameliorate overall quality of life. The KeNuT multistep dietary model should be integrated into a more comprehensive treatment plan, which may also include pharmacological interventions and bariatric surgery, depending on the severity of the disease and on patient’s compliance and acceptance.
